# Myeloperoxidase Polymorphism, Menopausal Status, and Breast Cancer Risk: An Update Meta-Analysis

**DOI:** 10.1371/journal.pone.0072583

**Published:** 2013-08-21

**Authors:** Xue Qin, Yan Deng, Zhi-Yu Zeng, Qi-Liu Peng, Xiu-Li Huang, Cui-Ju Mo, Shan Li, Jin-Min Zhao

**Affiliations:** 1 Department of Clinical Laboratory, First Affiliated Hospital of Guangxi Medical University, Nanning, Guangxi, China; 2 Department of Geriatrics, First Affiliated Hospital of Guangxi Medical University, Nanning, Guangxi, China; 3 Department of Orthopaedic Trauma Surgery, First Affiliated Hospital of Guangxi Medical University, Nanning, Guangxi, China; University of Ulm, Germany

## Abstract

Myeloperoxidase (MPO) is a metabolic/oxidative lysosomal enzyme secreted by reactive neutrophils at the sites of inflamed organs and tissues during phagocytosis. MPO has been either directly or indirectly linked to neoplasia, which is a well-established risk factor for many types of cancer. A large number of studies have reported the role of MPO G-463A polymorphism regarding breast-cancer risk. However, the published findings are inconsistent. Therefore, we conducted a meta-analysis to determine more precise estimations for the relationship. Eligible studies were identified by searching several electronic databases for relevant reports published before June 2012. According to the inclusion criteria and exclusion criteria, a total of five eligible studies were included in the pooled analyses. When the five eligible studies concerning MPO G-463A polymorphism were pooled into this meta-analysis, there was no evidence found for a significant association between MPO G-463A polymorphism and breast-cancer risk in any genetic model. We also categorized by ethnicity (Caucasian or Asian) for subgroup analysis; according to this subgroup analysis, we found no significant association between MPO G-463A polymorphism and breast-cancer risk in any genetic model. However, in the stratified analysis for the premenopausal group, women carrying the AA genotype were found to have a significantly reduced risk (OR = 0.56, 95% CI 0.34–0.94, p = 0.027). Under the recessive model, there was a significant association between MPO G-463A polymorphism and breast-cancer risk (OR = 0.57, 95% CI 0.34–0.93, p = 0.025). We conclude that MPO-G463A polymorphism might not be a good predictor of breast-cancer risk, though menopausal status modified women’s risk of developing breast cancer.

## Introduction

Breast cancer is the most common type of malignant neoplasm in women worldwide, and its incidence is increasing in both developed and developing countries [1]. Multiple genetic and environmental factors are known to be risk factors for breast cancer. Chemicals with carcinogenic potential, such as polycyclic aromatic hydrocarbons (PAHs) or aromatic amines, are common in the ambient environment, and certain PAHs have been identified as known or suspected human carcinogens [2]. Although genotoxicity occurs following a complex process of metabolic biotransformation, ultimate reactive species will form DNA adducts, which, if not repaired, lead to modifications of the genetic material [3].

Myeloperoxidase (MPO) is a metabolic/oxidative lysosomal enzyme secreted by reactive neutrophils at the sites of inflamed organs and tissues during phagocytosis [4]. MPO has been found in breast secretions as an antimicrobial enzyme, which is involved in DNA adduct formation through the activation of xenobiotics, such as PAHs and aromatic amines. These form chemically reactive oxygen species (ROS) in mammary epithelial cells [5]. A single nucleotide polymorphism (SNP) G-463A (rs2333227) is the most extensively studied polymorphism in MPOs and is located in the promoter region of the MPO gene. MPO A allele carriers are reported to confer lower mRNA expression and transcriptional activation than the 463 G common allele in vitro [6], while the G allele has been associated with increased MPO mRNA and protein levels in human monocyte-macrophages [7], [8]. Dally et al. [9] reported that MPO G-463A (G/A+A/A) genotypes were a protective factor in lung cancer patients; the same results were found for hepatoblastoma patients [10]. However, the GG genotype has been associated with high levels of MPO expression, which is correlated with acute promyelocytic leukemia [11]. These studies indicate that MPO-mediated oxidation exists in a wide variety of cancers, especially those involving chronic inflammation and/or prolonged neutrophil invasion.

Several studies have shown the possible involvement of MPO in the pathogenesis of breast cancer; however, the conclusions are inconsistent. To our knowledge, G-463A is the most extensively studied polymorphism in the MPO gene with respect to breast cancer susceptibility. We therefore undertook a meta-analysis to evaluate the association between MPO G-463A polymorphism and breast cancer risk.

## Materials and Methods

### Publication search

Eligible studies were identified by means of an electronic search of PubMed, Elsevier ScienceDirect, EMBASE, and EBSCO for relevant reports published before June 2012 with the following search terms: ‘‘myeloperoxidase’’ or ‘‘MPO’’ combined with ‘‘breast cancer.’’ All eligible studies were examined carefully. Review articles and references cited in the retrieved articles were manually obtained to find additional eligible studies.

### Inclusion and exclusion criteria

The following criteria were used for the study selection: the study (1) evaluated the association between MPO G-463A polymorphism and the risk of breast cancer; (2) used a case-control design; (3) included a full-text article; (4) offered the size of the sample and sufficient data (genotype distributions of both cases and controls were available) for estimating an odds ratio (OR) with a 95% confidence interval (CI) or information for helping infer the results in the papers; and (5) used the English language. Exclusion criteria were as follows: the study (1) used only case-group data, (2) included no available genotype frequency, and (3) used overlapping data published by the same first author.

### Data extraction

Based on the inclusion and exclusion criteria, the following data were extracted for each study: the first author’s surname, year of publication, ethnicity of the subjects, enrollment criteria of case and control, genotyping methods, study size, and genotype distribution in cases and controls (GG, GA, and AA genotypes for MPO G-463A polymorphism). Two authors from the present study (Qin and Deng) independently and carefully collected the data. For conflicting evaluation, these two authors carried out discussions until a consensus was reached. If they could not reach a consensus, disagreement was adjusted by a third author (Zeng).

### Methodological quality assessment

Three reviewers (Qin, Deng, and Zeng) independently evaluated the quality of selected studies by scoring according to a set of predetermined criteria (Table 1), which were modified from a previous meta-analysis of molecular association studies [12]–[15]. Scores ranged from 0 to 12, with higher scores indicating better quality. Disagreements were resolved by discussion.

**Table 1:** Scale for methodological quality assessment.

Criteria	Score
1.Representativeness of cases	
Consecutive/randomly selected from case population with clearly defined sampling frame	2
Consecutive/randomly selected from case population without clearly defined sampling frame or with extensive inclusion/exclusion criteria	1
Not described	0
2.Source of controls	
Population or community based	3
Hospital-based (cancer-free controls)	2
Hospital-based healthy volunteers without total description	1
Not described	0
3.Ascertainment of breast cancer	
Histopathologic confirmation	2
Diagnosis of breast cancer by patient medical record	1
Not described	0
4.pecimens of cases determining genotypes	
White blood cells or normal tissues	1
Tumor tissues or exfoliated cells of tissue	0
5.Sample size	
>1000	2
200–1000	1
<200	0
6.Quality control of genotyping methods	
Repetition of partial/total tested samples	1
Not described	0
7.Hardy-Weinberg equilibrium in control subjects	
Hardy-Weinberg equilibrium	1
Hardy-Weinberg disequilibrium	0

### Statistical analysis

The strength of association between MPOs and breast cancer risk was measured by ORs with 95% CIs. The pooled ORs were calculated, including (1) the GA genotype versus the GG genotype, (2) the AA genotype versus the GG genotype, (3) GG+GA genotypes versus the AA genotype (the dominant model), and (4) the GG genotype versus GA+AA genotypes (the recessive model). Heterogeneity between and within groups was checked by using the Q statistic. A value of p≥0.10 for the Q-test indicated there was no heterogeneity among studies, so the pooled OR estimate from each study was calculated by the fixed-effects model; otherwise, the random-effects model was used [16], [17]. A funnel plot was carried out in order to estimate potential publication bias. Funnel-plot asymmetry was assessed using Egger’s linear-regression test, a linear-regression approach for measuring funnel-plot asymmetry on the natural logarithmic scale of the OR. In the control populations, Hardy–Weinberg equilibrium (HWE) was evaluated using the goodness-of-fit chi-square test. A value of p<0.01 signified a departure from HWE. Sensitivity analysis was performed to evaluate the stability of the results by removing the studies not in Hardy–Weinberg equilibrium. All the statistical tests were performed with STATA version 10.0 (Stata Corporation, College Station, TX).

## Results

### Characteristics of studies

**Figure 1:**
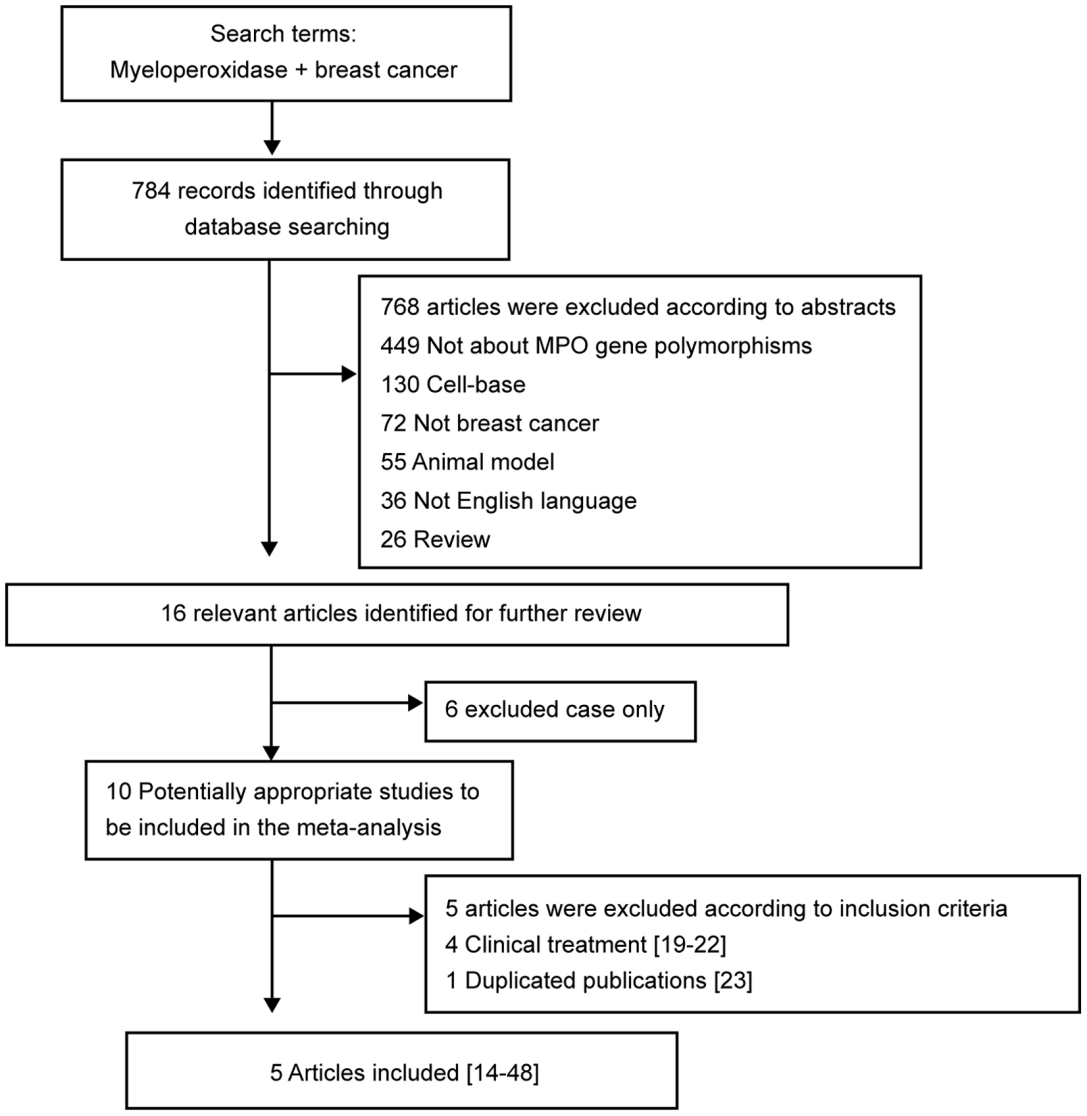
Flow chart of meta-analysis.

Five eligible studies were included in the pooled analyses (Figure 1) [18]–[22], and their characteristics are summarized in Table 2. We also included one study that investigated the T-764C (rs2243828) polymorphism, given its 100% genotype concordance with G-463A polymorphism in Caucasians (http://snp500cancer.nci.nih.gov). Four clinical treatment studies were excluded [23]–[26]. In two studies [20], [27] that used the same study participants, we selected the most recent study [20]. There were three studies of Caucasian populations [18], [20], [21] and two of Asian populations [19], [22]. Three studies reported the effects of MPO-G463A polymorphism in premenopausal and postmenopausal women [18], [20], [21], and we analyzed the premenopausal and postmenopausal subgroups separately (Table 3).

**Table 2:** Characteristics of studies included in the meta-analysis.

First author	Quality score	Year	HWE	Ethnicity	Enrollment criteria		Genotyping methods	Study size	Genotypes distribution (case/control)		
					Case	Control		Case /Control	GG	GA	AA
Ahn[18]	10	2004	0.034	Caucasion	newly diagnosed breast cancer cases	matched population	MALDI-TOF	1011/1067	630/632	321/362	60/73
Lin[19]	9	2005	0.013	Asian	Pathologically confirmed breast carcinoma cases	matched to each case by age (±2 years), residence, and date of blood sample collection (±3 months).	PCR-RFLP	99/366	76/285	20/70	3/11
Li[20]	10	2009	0.212	Caucasion	identified breast cancer cases	matched to cases on age (±6 months), race/ethnicity (White, African-American, Hispanic, Asian and other/unknown) and date of blood collection (±6 months).	TaqMan	417/403	245/250	153/140	19/13
He[17]	12	2009	0.987	Caucasion	Histopathologic characteristics of breast tumors cases	matched to cases on year of birth, menopausal status, recent post-menopausal hormone use, month of blood return, time of day of blood collection, and fasting status at blood draw.	TaqMan	1209/1678	762/1062	405/546	42/70
Tsai[22]	8	2012	0.013	Asian	199 Ductal carcinoma, 29 Lobular carcinoma, and 32 Other neoplasms cases	Non-smoking and non-drinking women, without a present or previous history of breast cancer	Real-time PCR	260/224	174/132	86/88	0/4

HWE Hardy–Weinberg equilibrium.

**Table 3:** Characteristics of studies stratified by menopausal status.

		Pre-menopausal	Post-menopausal
		Genotypes distribution (case/control)	Genotypes distribution (case/control)
First author	Year	GG GA AA	GG GA AA
Ahn[18]	2004	205/210 108/117 19/35	408/395 208/231 40/36
Li[20]	2009		245/250 153/140 19/13
He[17]	2009	154/196 81/90 6/13	527/773 292/414 33/52

### Meta-analysis

In the five studies of MPO G-463A polymorphism, we identified 2996 cases and 3738 controls. Overall, the results showed no significant association between MPO G-463A polymorphism and breast cancer risk (GA versus GG: OR = 0.97, 95% CI 0.87–1.08; AA versus GG: OR = 0.87, 95% CI 0.68–1.10; dominant model: OR = 0.96, 95% CI 0.87–1.06; recessive model: OR = 0.88, 95% CI 0.69–1.11) (Table 4). In the subgroup analysis by ethnicity, we found no significant association in any of the genetic models (Table 4).

**Table 4:** Genetic polymorphism of MPO and breast cancer risk.

Genetic model	Ethnicity	No. of studies	OR	95%CI	*P*	Statistical model	*I^2^* (%)	*P_ h_* a
GA vs GG	Asian	2	0.83	0.61–1.13	0.242	Fixed	13.5	0.282
	Caucasian	3	0.99	0.89–1.11	0.870	Fixed	9.2	0.333
	All	5	0.97	0.87–1.08	0.582	Fixed	10.2	0.348
AA vs GG	Asian	2	0.53	0.17–1.64	0.267	Fixed	60.1	0.114
	Caucasian	3	0.89	0.69–1.14	0.357	Fixed	9.4	0.332
	All	5	0.87	0.68–1.10	0.242	Fixed	15.6	0.315
GA+AA vs GG	Asian	2	0.81	0.60–1.10	0.178	Fixed	34.5	0.217
	Caucasian	3	0.98	0.88–1.09	0.685	Fixed	29.8	0.241
	All	5	0.96	0.87–1.06	0.405	Fixed	29.5	0.225
AA vs GG+GA	Asian	2	0.54	0.17–1.68	0.285	Fixed	55.9	0.132
	Caucasian	3	0.90	0.70–1.15	0.393	Fixed	0.0	0.396
	All	5	0.88	0.69–1.11	0.275	Fixed	4.1	0.383

a *P* value for heterogeneity based on Q test.

**Figure 2:**
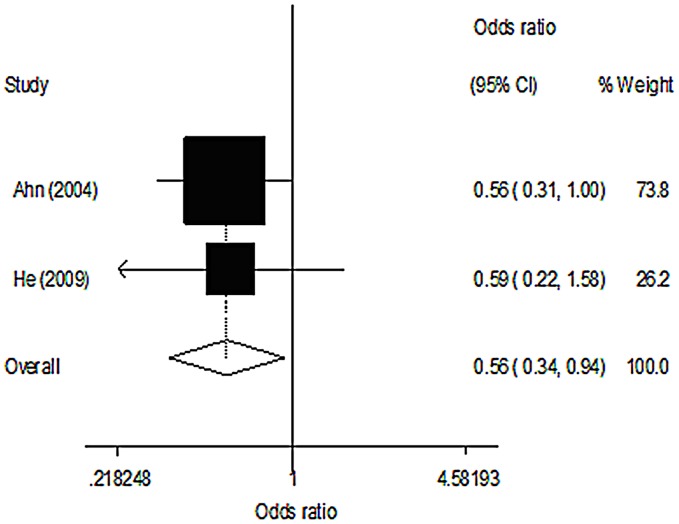
Meta-analysis of OR for MPO polymorphism associated with breast cancer in pre-menopausal women (AA versus GG).

**Figure 3:**
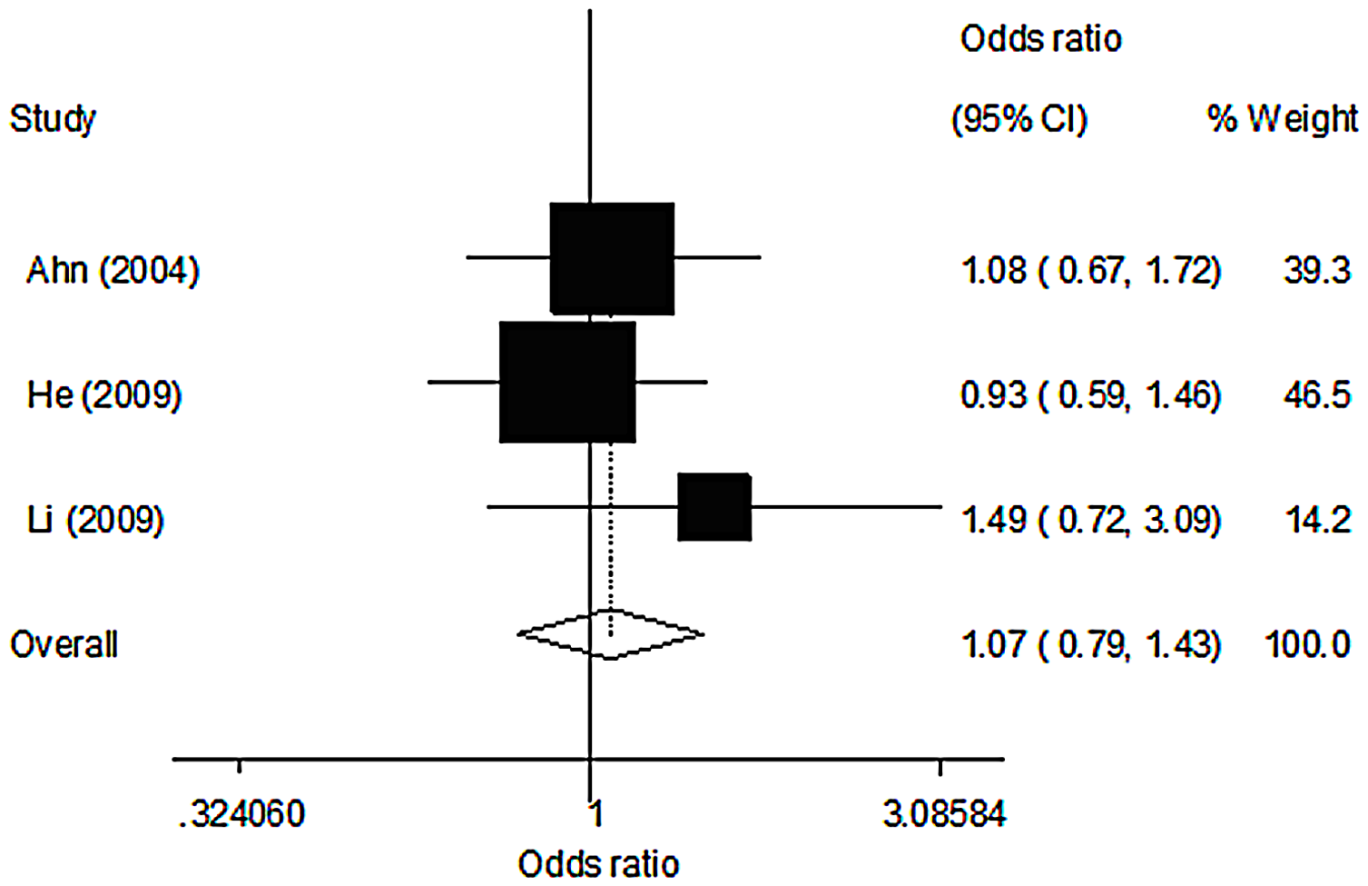
Meta-analysis of OR for MPO polymorphism no associated with breast cancer in post-menopausal women (AA versus GG).

The premenopausal (573 cases, 661 controls) and postmenopausal (1925 cases, 2304 controls) groups came from three and two studies, respectively. In the premenopausal group, women carrying the AA genotype were found to have a significantly reduced risk of breast cancer (OR = 0.56, 95% CI 0.34–0.94, p = 0.027), and we found a significant association between MPO G-463A polymorphism and breast cancer risk with the recessive model (OR = 0.57, 95% CI 0.34–0.93, p = 0.025) (Table 5, Figure 2). In the postmenopausal group, no significant association was observed in any of the genetic models (Table 6, Figure 3).

**Table 5:** Genetic polymorphism of MPO and breast cancer risk in pre-menopausal women.

Genetic model	OR	95%CI	*P*	Statistical model	*I^2^* (%)	*P_ h_* a
GA vs GG	1.03	0.81–1.31	0.820	Fixed	0.0	0.443
AA vs GG	0.56	0.34–0.94	0.027	Fixed	0.0	0.926
GA+AA vs GG	0.94	0.75–1.19	0.616	Fixed	0.0	0.339
AA vs GG+GA	0.57	0.34–0.93	0.025	Fixed	0.0	0.987

a *P* value for heterogeneity based on Q test.

**Table 6:** Genetic polymorphism of MPO and breast cancer risk in post-menopausal women.

Genetic model	OR	95%CI	*P*	Statistical model	*I^2^* (%)	*P_ h_* a
GA vs GG	1.00	0.87–1.13	0.949	Fixed	0.2	0.367
AA vs GG	1.07	0.79–1.43	0.665	Fixed	0.0	0.557
GA+AA vs GG	1.00	0.89–1.14	0.944	Fixed	0.0	0.394
AA vs GG+GA	1.08	0.80–1.44	0.627	Fixed	0.0	0.570

a *P* value for heterogeneity based on Q test.

### Sensitivity analysis

**Figure 4:**
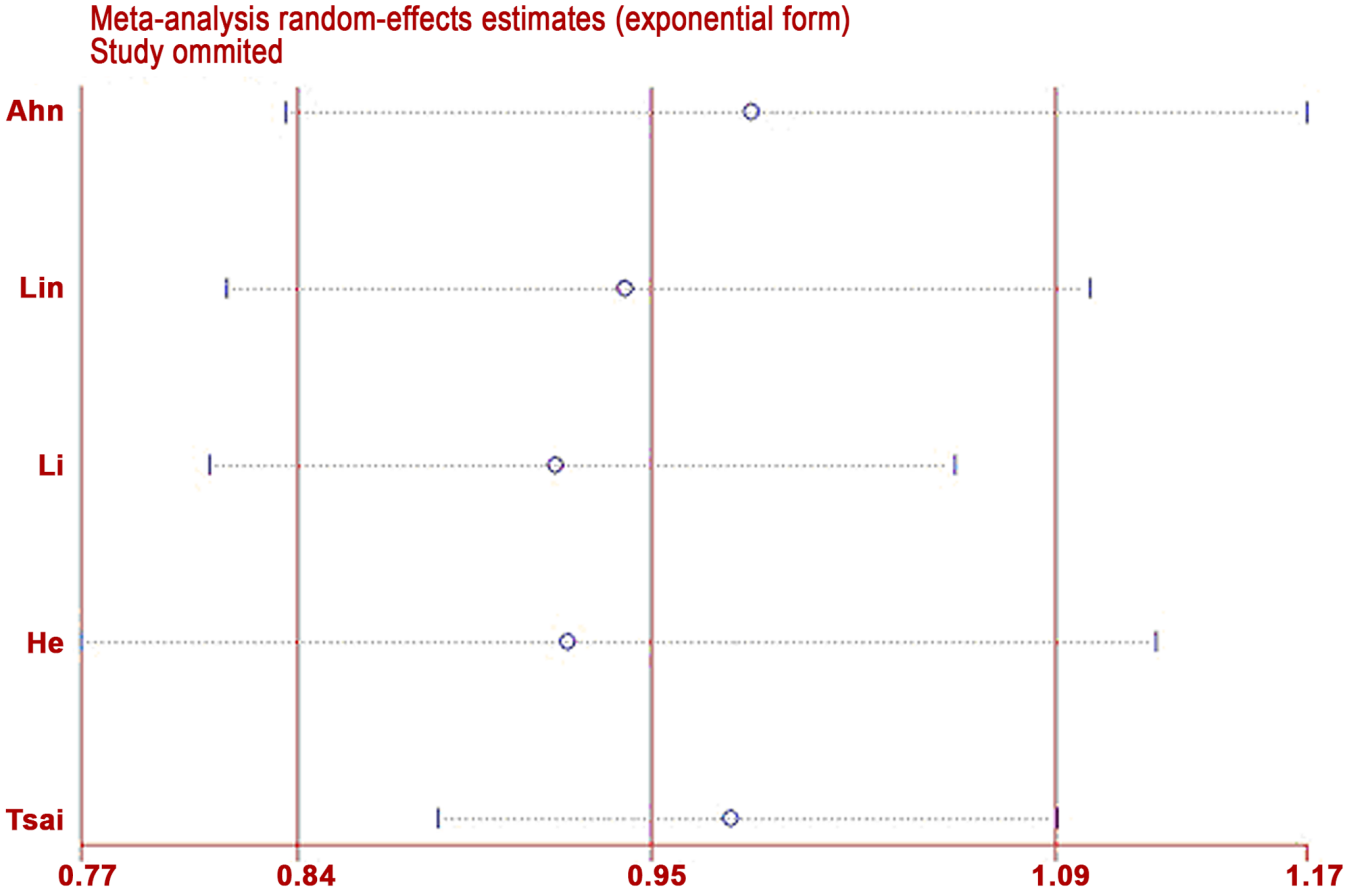
Sensitivity analysis through deletion of one study at a time to reflect the influence of the individual dataset to the pooled ORs in GA+AA versus GG.

A single study involved in the meta-analysis was deleted each time to reflect the influence of the individual data set to the pooled ORs. The results suggested that no individual study significantly affected the pooled ORs. Sensitivity analysis was performed after excluding HWE-violating studies, and the corresponding pooled ORs were not materially altered, indicating that our results are statistically robust (Figure 4).

### Publication bias

**Figure 5:**
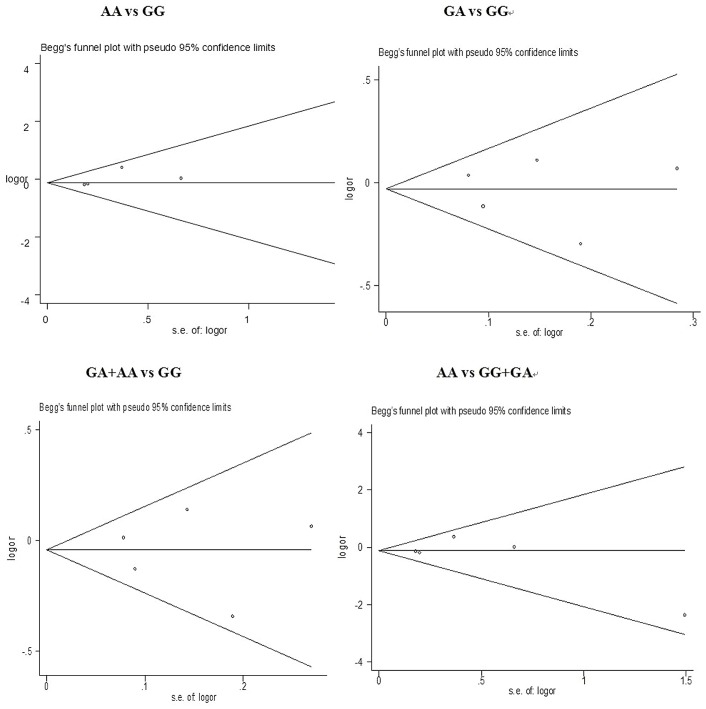
Begg’s funnel plot with pseudo 95% CI of publication bias test for MPO polymorphism. Each point represents a separate study for the indicated association. Log[OR] natural logarithm of odds ratio.

Both Begg’s funnel plot and Egger’s test were performed in order to assess the publication bias of the literature. As shown in Figure 5, Begg’s funnel plots did not reveal any evidence of obvious asymmetry in any of the comparison models in the overall meta-analysis. The results of the Egger’s test also did not show any evidence of publication bias (Table 7). However, based on the recommendations of the Cochrane Handbook for Systematic Reviews of Interventions (www.cochranehandbook.org), which states that the test for publication bias yields unreliable results when less than 10 studies are included in a meta-analysis, the negative results in our meta-analysis are possibly because the number of publications we assessed were too few to determine statistical significance.

**Table 7:** Publication bias tests for comparisons involving the MPO polymorphism.

Genetic comparision	Coefficient	Standard error	t	*P*	95% CI
AA vs GG	–0.3721	1.049	–0.35	0.746	–3.713–2.968
GA vs GG	–0.3639	1.369	–0.27	0.808	–4.723–3.995
GA+AA vs GG	–0.2676	1.543	–0.17	0.873	–5.178–4.643
AA vs GG+GA	–0.4005	0.974	–0.41	0.709	–3.502–2.701

## Discussion

MPO is a member of the mammalian heme peroxidase enzyme family and is a key component of the phagocyte oxygen-dependent intracellular microbicidal system, playing an important role in innate immune responses [28]. MPO has been directly and indirectly linked to neoplasia and is involved not only in the production of oxidative hypochlorous acid from H_2_O_2_ during infection but also in the metabolic activation of a number of procarcinogens [9], which are known to be risk factors for many types of cancer. Feyler et al. [29] reported that carriers of the G/A genotype with a reduced risk of bladder cancer compared with the subjects with the MPO G/G genotype, (OR =  0.5, 95% CI 0.29–0.88). Hung et al. [30] found that the MPO G-463A homozygous variant was associated with a reduced risk of bladder cancer (OR = 0.31, 95% CI 0.12–0.80). The A allele has been show to be associated with a 50% reduced risk of hepatoblastoma (OR = 0.51, 95% CI 0.27–0.93), and the G/A or A/A genotype reduced the risk of hepatoblastoma by 56% (OR = 0.44, 95% CI 0.21–0.90) [10]. Numerous studies have reported the role of MPO G-463A polymorphism in affecting the risk of breast cancer, but results differ and the genetic linkages have not always been replicated. Hence, we conducted a meta-analysis to explore the association between MPO G-463A polymorphism and breast cancer risk.

Unlike studies that have noted an association between the MPO A/A genotype and a reduced risk of breast cancer, we did not find an association between MPO G-463A polymorphism and breast cancer risk; however, consistent with the findings of Lin et al. [19], we did find a significantly reduced risk (OR = 0.56) in premenopausal women carrying the AA genotype. Reynolds et al. [31] showed that the MPO GG genotype is associated with an increased incidence of Alzheimer's disease in females and decreased incidence in males, which could be attributed to the effects of sex hormones on MPO gene expression. In addition, Békési documented that intracellular myeloperoxidase activity in neutrophils was lower in postmenopausal women than in premenopausal women [32]. Our meta-analysis findings supported the potential role of estrogen in the regulation of MPO activity.

We have read with great interest the recent meta-analysis reported by Pabalan et al [33]. The data reported by Pabalan et al. [33] regarding the study of Li et al.[20] do not seem in line with the data provided by Li et al [20] in their original publication. The numbers reported by Li et al. [20] under age-adjusted model, in cases and controls, are 477 and 462. While, under fully adjusted model, in cases and controls, are 417 and 403. Interestingly enough, after carefully studying the data presented by Pabalan et al. [33], the numbers in cases and controls, are 894 and 865..In our opinion, it was inappropriate to combinate the results since different models were used and combination resulted in misrepresentation of the original data. Therefore, when we extracted the data from Li et al. [20] for our meta-analysis, we only adopted the data using the fully adjusted model (417 cases and 403 controls) in order to ensure that the ethnicity of the participants was Caucasian. Chu et al. [34] reported in another meta-analysis that there was no association between MPO-G463A polymorphism and breast cancer, regardless of the menopausal status and ethnic background. Although our results are not fully accord with previous meta-analysis regarding the positive association for MPO-G463A polymorphism polymorphism, we have made much more powerful and detailed analysis to support our findings, which made our results much more reliable compared with previous meta-analysis: (1) we included more studies; (2) our analysis involved further subgroup analysis stratified according to Asian and Caucasian ethnicity, which contribute to decrease the geographical heterogeneity of the included studies, but the previous meta-analysis did not do; (3) we considered the association between menopausal status and breast cancer which previous meta-analysis also did not do; (4) we were more rigorous than previous meta-analysis in extracting data especially from the study by Li et al..[20],

However, there were also several limitations to our study. First, our meta-analysis had a limited statistical power for subgroup analysis, and the precision of our estimates needs to be assessed in consideration of the small numbers. Our analysis of premenopausal women only included two studies, both of which were of Caucasian women. Clarifying whether ethnicity has a biological influence on cancer susceptibility, therefore, requires more subjects and different ethnicities. Second, as positive associations have a greater chance of publication than true negatives, many studies remain unpublished and were therefore not included in this meta-analysis. Finally, due to a lack of original data, such as age, alcohol consumption, and other factors, we could not perform a more precise calculation of adjusted ORs, which limited our analysis of potential gene-environment interactions.

## Conclusions

In summary, our results indicated that MPO-G463A polymorphism might not be a good predictor of breast cancer risk, while menopausal status modifies a woman’s risk of breast cancer. Premenopausal women carrying the AA genotype were found to have a decreased risk of breast cancer. However, given the limited data, more studies are needed to clarify the role of MPO in breast carcinogenesis.

## Supporting Information

Text S1 **Checklist**
(DOC)
